# Polygenic Risk Scores Shed Light on the Relationship between Schizophrenia and Cognitive Functioning: Review and Meta-Analysis

**DOI:** 10.3390/jcm9020341

**Published:** 2020-01-25

**Authors:** Jasmina Mallet, Yann Le Strat, Caroline Dubertret, Philip Gorwood

**Affiliations:** 1 APHP; Department of Psychiatry, Universitary Hospital Louis Mourier, 92700 Colombes, France; yann.lestrat@aphp.fr (Y.L.S.); caroline.dubertret@aphp.fr (C.D.); 2 Université de Paris, Institute of Psychiatry and Neuroscience of Paris (IPNP), INSERM U1266, F-75014 Paris, France; 3 GHU Paris Psychiatrie et Neurosciences, Hôpital Sainte Anne, F-75014 Paris, France

**Keywords:** schizophrenia, cognition, intelligence, educational attainment, genome wide association study (GWAS), genetics, polygenic risk score

## Abstract

Schizophrenia is a multifactorial disease associated with widespread cognitive impairment. Although cognitive deficits are one of the factors most strongly associated with functional impairment in schizophrenia (SZ), current treatment strategies hardly tackle these impairments. To develop more efficient treatment strategies in patients, a better understanding of their pathogenesis is needed. Recent progress in genetics, driven by large genome-wide association studies (GWAS) and the use of polygenic risk scores (PRS), has provided new insights about the genetic architecture of complex human traits, including cognition and SZ. Here, we review the recent findings examining the genetic links between SZ and cognitive functions in population-based samples as well as in participants with SZ. The performed meta-analysis showed a negative correlation between the polygenetic risk score of schizophrenia and global cognition (*p* < 0.001) when the samples rely on general and healthy participants, while no significant correlation was detected when the three studies devoted to schizophrenia patients were meta-analysed (*p* > 0.05). Our review and meta-analysis therefore argues against universal pleiotropy for schizophrenia alleles and cognition, since cognition in SZ patients would be underpinned by the same genetic factors than in the general population, and substantially independent of common variant liability to the disorder.

## 1. Introduction

### 1.1. Cognition and Schizophrenia: Is Genetic the Missing Link?

In the general population, cognitive functioning is positively associated with greater longevity and less physical and psychiatric morbidity, and negatively associated with several quantitative disease risk factors and indices [1]. Higher general cognitive function in childhood is predictive of lower self-reported psychological distress decades later [2]. This association between cognitive function and psychological well-being extends to severe psychiatric conditions. As an example, a standard deviation lower score in IQ is associated with a 60% increased risk for hospitalization for schizophrenia, a 50% increase for mood disorders, and a 75% greater risk for alcohol-related disorders in a two decades follow-up [3]. Lower scores on standard tests of intelligence have consistently been associated with an increased risk of schizophrenia [4].

It is not clear whether schizophrenia affects cognitive functioning or vice versa, or whether both are influenced by some common factors. The associations between cognitive functioning and schizophrenia may, in part, reflect shared genetic influences.

### 1.2. Schizophrenia is a Partly Genetic and Cognitive Disease

Schizophrenia is a severe and chronic psychiatric disorder that affects up to 1% of the worldwide population. It is among the leading causes of disability worldwide. Schizophrenia is a multifactorial disease, albeit highly heritable (*h*^2^ = 0.6–0.8) [5,6]. Schizophrenia (SZ) is influenced by many genetic variants, each probably having a small effect. The last decade brought large progress in the field of schizophrenia genetics. The largest genome-wide association study (GWAS) to date has identified 108 loci harbouring common risk variants, likely to contribute to SZ risk [7]. While each single nucleotide polymorphism (SNP) carries only a subtle increase in schizophrenia risk (with odds ratios in the range of 1.1 to 1.2), their combination into a polygenic risk score (PRS) provides a stronger predictor of disease [8]. High-penetrance genetic variations also contribute to the genetic risk for schizophrenia (please refer to [9] for review).

SZ diagnosis relies on the presence of positive and negative symptoms, but cognitive dysfunction is now regarded as a core component of the disorder rather than a consequence of the disease or an adverse effect of medication. Compared with healthy individuals, patients with schizophrenia display widespread cognitive impairments including deficits in learning, memory, processing speed, attention, and executive functioning [10,11,12,13]. Cognitive dysfunction often precedes the onset of psychosis by several years and seems to remain stable over the course of the disease [14]. It is also one of the most robustly replicated intermediate phenotypes in schizophrenia. Neurocognitive measures of working memory, verbal declarative memory, and sustained attention are particularly salient endophenotypes for schizophrenia, given the prominent deficits in these cognitive domains observed in patients and, to a lesser extent, their unaffected relatives [15]. Thus, it has been argued that studying cognitive traits could help in understanding the etiology of SZ [16]. 

While these cognitive impairments are heritable features of SZ (*h*^2^ = 0.20–0.80) [17], little is known on contributing genes. Twin and family studies have shown that the liabilities to cognitive dysfunction and schizophrenia covary [18]. Unaffected relatives of patients present cognitive dysfunction, and healthy first-degree relatives in multiplex families (with a least two relatives having the same disorder within the same family) are more impacted by cognitive impairment then those in simplex family [19]. These results suggest that genetic risk for schizophrenia could contribute to cognitive impairment. This statement was corroborated by the cognitive, genetic and MRI examination of a large genotyped sample of control subjects, in which authors found an intermediate phenotype (cognitive and MRI) in controls carrying copy number variants which were labelled as “at risk” [20].

### 1.3. The Genetic Aspects of Cognitive Functioning 

Cognitive functions are now also regarded as a combination of genetic and environmental factors, with the general cognitive function (so called “g”) being highly heritable and polygenic. Twin studies have contributed to improve our knowledge on the heritability of cognitive functions and intelligence [21]. Heritability of cognitive functions is estimated as ranging between *h*^2^ = 0.33 and *h*^2^ = 0.80 [22], with a difference for intelligence between childhood (*h*^2^ = 0.45) and adulthood (*h*^2^ = 0.80) [23].

Recent data have largely improved the characterization of genetic factors involved in cognitive functioning with the use of polygenic scores. Polygenic scores are sometimes referred as polygenic risk score (PRS) or genome-wide polygenic scores (GPS). The latest versions avoid the term ‘risk', as used in the polygenic risk score, which implies that genetic influences are inevitably associated with negative outcomes, while recently it has been associated with resilience factors [24]. In non-clinical cohorts, GWAS has been useful to better characterize the genetic component of cognition. These studies have also shed light on genetic correlations between SZ and cognition [25,26].

Most of these studies use the “g approach”. The g factor represents the general cognitive function and is defined as a latent trait underlying shared variance across multiple subdomains of cognition. More details and a summary of GWAS of general cognitive function can be found in [22]. Some specific cognitive domains are also highly heritable [27]. In a meta-analysis of 170 published twin and family heritability studies of more than 800,000 non-psychiatric and schizophrenia subjects, authors estimated heritability across many neuropsychological tests and cognitive domains [17]. Heritability ranged across phenotypes, likely due to differences in genetic and environmental effects, with the highest heritability for general cognitive ability (32–67%), verbal ability (43–72%), visuospatial ability (20–80%), and attention/processing speed (28–74%), while the lowest heritability was observed for executive function (20–40%). These results confirm that many cognitive phenotypes are under strong genetic influences. Heritability estimates were comparable in non-psychiatric and schizophrenic samples, despite differences in environmental factors and illness-related moderators (differences in ascertainment, medication…). They also found a genetic overlap between cognitive phenotypes and schizophrenia liability in twin studies, suggesting partially shared genetic aetiology. This similar heritability of cognitive deficits in SZ samples and nonclinical samples suggests that studying schizophrenia samples is valuable and more informative for understanding the relationship between cognition and schizophrenia, than studying unaffected relatives. This is an important issue because it is commonly thought that environmental factors and illness-related moderators hinder the detection of genetic effects in schizophrenia samples [17].

### 1.4. Why Investigate the Genetic Link between Cognition and Schizophrenia?

The idea encapsulated in recent studies is that the discovery of genetic loci implicated in the modulation of cognitive abilities in young adulthood, may shed light into neuronal processes and the underlying molecular mechanisms that, when compromised, increase the risk to develop SZ. Moreover, cognition is a particularly important endophenotype because discovering its underlying genetic mechanisms may help identify promising targets for improving cognitive functioning in patients. Indeed, there are already evidence that genetics might help predict cognitive responses to drugs in schizophrenia [28,29]. GWASs that examine millions of genetic variants are a powerful tool to identify common variants responsible for susceptibility to common and complex diseases. The Psychiatric Genomics Consortium (PGC) is the largest GWAS to date concerning schizophrenia (using 36,989 patients and 113,075 controls). The PGC identified 108 loci including genes and genetic variants related to schizophrenia. Several GWAS have been performed on large population based samples, including cognitive data, with different methods (general intelligence factors, subtests, educational attainment). The largest ones have found more than 100 genome-wide significant loci related to cognitive function [30,31].

### 1.5. Cognitive Functioning and Community Functioning in Schizophrenia

Lastly, although cognition is one of the most important predictors of community functioning in schizophrenia [32,33], little is known about the causes of this correlation [34]. Integrated interventions targeting both neurocognition (and social cognition) may optimally improve functional outcomes. Thus, the need to treat these impairments has become a priority. Studies of emerging mechanisms for treating cognitive impairments suggest that they are somewhat modifiable through both pharmacological and psychological intervention [35]. However, current treatment strategies largely fail to ameliorate these cognitive impairments, therefore leading to little efficacy on functioning. To develop more efficient treatment strategies in patients with schizophrenia, a better understanding of the specific cognitive processes involved and the pathogenesis of these cognitive deficits is needed. In SZ patients, genetics would specifically explain the large association between cognition and community functioning. Indeed, a large study (on 43 families, each with at least two SZ patients and 135 controls), authors found that genetic effects are shared between cognition and functioning in schizophrenia, but not with another psychiatric diagnosis (major depression) or in healthy individuals [36].

In conclusion, accumulating evidence indicates that genetic risk of schizophrenia may contribute to cognitive dysfunction and reciprocally [22,37]. Other data indicate a strong and specific genetic link between cognitive impairment and loss of functioning [36], yet with few data to date. Recent findings suggest that the genetic risk of schizophrenia may differentially impact cognition in patients versus not, shedding an insight on the underpinning of cognitive impairment related to schizophrenia [28,29]. However, no study has provided a meta-analytic approach of this differential effect.

In this work we will (i) review and discuss to what extent the link between cognitive functioning and schizophrenia is underpinned by genetics according to recent studies using GWAS and particularly PRS, (ii) analyse data to provide a meta analytic approach of the genetic correlation between general cognitive functioning and genetic risk of schizophrenia in participants with schizophrenia and in controls.

## 2. Experimental Section

### 2.1. Review 

Medline searches were performed to review the literature from the last 5 years (until November 2019), and the following keywords were used: (schizophrenia OR psychotic disorder) AND (cognition OR educational attainment OR intelligence) AND (GWAS OR Polygenic Risk Scores).

We chose to include only the most up to date publications, as the first large genetic consortium in schizophrenia was published in 2014 [7]. However previous relevant studies (even if not reported in Table S1) or reviews on this topic are also discussed throughout this work.

A total of 254 results was identified. We then selected studies published in English that included only human participants with an abstract available, which restricted the results to *n* = 127. After exclusion of reviews and expert opinions, duplicates or non-related topics, 27 papers were examined. We then added all relevant papers through the references of the above-mentioned papers, which rose the examined papers to 40.

### 2.2. Meta-Analysis

We identified relevant papers from our review for determining the correlation between SZ PRS and global cognitive functioning in subjects with schizophrenia versus other subjects. Studies concerning children or elder adults were excluded, as well as studies providing data for cognitive subtests only. Whenever available, data required for effect size determination were extracted and treated by the first author (JM). In order to facilitate comparisons across studies that have used different measures of effect size, we used a table of equivalencies for the three most common measures (ROC area (AUC) or z, Cohen’s d, and r. [38]. Effect sizes were computed as the Pearson’s correlation coefficient r. For greater accuracy and values beyond the table, we calculated r from z with *r* = (e^2Z^ − 1)/(e^2Z^ + 1).

Authors were contacted to provide data whenever possible (e.g., unpublished data by [39] were used). All data from [40] were analysed with a meta-regression of the correlation coefficient from each different COGENT datasets (data available on request). We finally included 6 studies concerning general and healthy populations, and 3 studies with participants with schizophrenia. Statistical analysis were made with Jamovi and MAJOR [41], WK (2019) (MAJOR: Meta-Analysis for JAMOVI, R package version 1.0.2, https://github.com/kylehamilton/MAJOR)

We conducted a basic meta-analysis of correlation coefficients using a Hunter and Schmidt approach, (what Hunter and Schmidt call a "bare-bones meta-analysis") [42]. This approach provides a random effect model of meta-analysis and is more appropriate in the psychiatric field. Indeed, Type I errors may be quite frequent in the meta-analysis literature in some research areas when researchers use fixed effects methods and interpret CIs as significant tests [43].

To perform such an analysis, three adjustments are necessary and possible with the Jamovi R package. In particular, we used an adjusted method for calculating the sampling variances of the correlation coefficients: the large-sample equation for the sampling variance of a (Pearson product-moment) correlation coefficient is Var (r_I_) = (1 − ρ_i_^2^ )^2/^(n_i_ − 1) (although one could also just divide by n_i_ and not n_i_ -1). Since this equation involves the (unknown) true correlation coefficient ρ_i_, it is not usable in practice. One approach is to substitute r_i_ for ρ_i ,_ but the resulting estimator Var (r_i_) can be very inaccurate, especially when the sample size of a study is small. Hunter and Schmidt instead suggest to calculate the sample-size weighted average of the correlation coefficients, that is: *r* = Σn_i_ r_i/_Σn_i_; and then to substitute r into the equation for the variance.

Then we used the sample sizes as weights in the analysis, and switched to the Hunter and Schmidt estimator to estimate the amount of heterogeneity. Various corrections are possible with the Hunter and Schmidt method. For example, due to measurement error in one or both variables of interest, the observed correlation coefficients tend to be attenuated (i.e., lowered/diluted). We thus applied the correction for attenuation to the observed correlations and we also corrected the sampling variances accordingly. Hunter and Schmidt recommend giving more weight not only to studies with larger sample sizes, but also studies that have higher reliability (or more generally, where the artifact correction factor is closer to 1 (the reader can refer to [42] and metafor-project.org for more details). 

We used funnel plot to evaluate publication biases and Rosenthal fail-safe’N method to test the robustness of the results. We estimated the extent of heterogeneity with several methods (namely the Q-statistic (also referred to as “Cochrane’s Q”); *I*^2^ as a measure for the proportion of observed variance that reflects real differences in effect size and both T^2^ and Tau as measures of the dispersion of true effect sizes between studies in terms of the scale of the effect size. Forest plots were also provided.

## 3. Results

### 3.1. Review

The main results of the review are listed in Table S1 (Supplementary Material).

#### 3.1.1. Implication of Genetic risk of Schizophrenia on Cognitive Functioning/Educational Attainment (EA)

Accumulating evidence indicates that genetic risk of schizophrenia may contribute to cognitive dysfunction [44], suggesting that cognitive deficit is reliably linked with inherited risk for schizophrenia and that it predates the diagnosis [17]. The MATRICS cognitive consensus battery (MCCB) group has identified six core cognitive domains that need to be enhanced in clinical trials for schizophrenia. These domains were all negatively correlated to higher genetic risk of schizophrenia in a recent study on healthy controls [45], corroborating previous findings on specific subtests on population based samples [46,47] and on a sample of older adults [48]. However, others failed to show a negative association between SZ PRS and cognitive subdomains [37,49].

Several studies examined the educational attainment (EA), generally represented by the number of years of education, and considered as a proxy of general cognition, being strongly correlated with general intelligence (“fluid intelligence”) [26]. EA is also polygenic and heritable [27]. It is also well recognized that lower educational attainment is associated with adverse health outcomes [50]. It has been used in earlier studies and, high EA is associated with lower schizophrenia risk whereas low EA with poorer outcomes [22]. However, the recent advances in genetics brought new paradoxical findings. A study found a higher propensity to succeed in the “English school level” and less attention to detail (belonging to “autism quotient”) in adolescents with high PRS [51], which could be in line with the propensity for creativity predicted by SZ PRS [52]. In another study, the genetic variants associated with obtaining a college degree were also related to higher genetic risk of schizophrenia [47], while genes related to SZ were negatively related to all cognitive tests. The authors hypothesized that the discrepancy between the cognitive and educational results is mediated by the age of the participants—if schizophrenia genes are detrimental to cognitive functioning only later in life, they may have differential effects on educational attainment (which tends to peak before age 30), and the cognitive tests (which were taken later). Other studies, with different methods, also report this positive correlation between the genetic risk for schizophrenia and EA. This could be due to SZ genetic link with bipolar disorder (BD) (positively correlated to EA) [53]. However, based on recent genetic analysis on EA and SZ, some authors have recently proposed the hypothesis that SZ patients aggregate over at least two disease subtypes: one part resembles high intelligence and bipolar disorder (BD), while the other part is a cognitive disorder that is independent of BD [54]. This could explain the discrepancy revealed by last year genetic findings on positive correlation between EA and SZ. Indeed, all current GWAS in schizophrenia are based on clinical manifestations, but not cognitive functions.

Some studies failed to show an impact of SZ PRS on general cognition. The finding that the polygenic component associated with schizophrenia is associated with only older age general cognitive function [55,56] could indicate that it is the variants that are protective of age-related cognitive decline, rather than the variants responsible for the development of cognitive function, that drive the genetic correlation between cognitive function and schizophrenia. Findings are however inconsistent, as SZ PRS were associated with decreased cognitive function (subtests) but not with cognitive decline in another study [48]. In this study, authors tested whether polygenic risk for a number of psychiatric disorders was associated with decreased general cognitive function in older adults, and whether this effect increased with age. They found that PRS specific for SZ (and neither BD nor Major Depressive Disorder) was associated with decreased total cognition scores, and that most of this association was driven by decreased performance on a subcomponent of the cognitive score measuring attention and language. Interestingly, the SZ PRS was not associated with greater cognitive decline (contrary to Alzheimer PRS). This highlights the potential differences of pathophysiology of cognitive deficits in SZ and Alzheimer (static effect of SZ risk alleles on cognition, schizophrenia being now considered as a neurodevelopmental disorder). Other studies converge toward this static effects of SZ risk alleles on cognition [37,40].

A study using the cFDR method (conditional False Discovery Rate), found three loci with the same effect directions in SZ and cognitive function, indicating that some SZ risk variants may improve cognitive function. This may help explaining why some patients with SZ show normal cognitive function [44]. Indeed the cFDR method allows identifying polygenic effects with opposite directions, contrary to standard methods (e.g., LD regression) that provides global positive, null or negative correlations between two traits. However, for 18 loci, SZ risk alleles were associated with poorer cognitive performance. Another study pointed a positive effect of SZ PRS on cognition [57].

The association between higher SZ PRS and better cognition was more noticeable when removing participants with medical conditions. The positive correlation between PRS and cognitive ability being stronger in the healthiest end of the phenotypic distribution would be in concordance with an interplay between resilience and genetic schizophrenia risk [57].

Finally, studies on young population sample bring divergent results, sometimes on the same samples, but converge toward an early association between genetic liability to SZ and cognitive impairment [46,58,59].

#### 3.1.2. Implication of Cognition/EA on the Risk for Schizophrenia and Correlations

Few studies directly explore the implication of cognitive PRS on the risk for schizophrenia. One of the first study applying polygenic SNP score derived from a large-scale cognitive GWAS cognitive meta-analysis (issued from non-clinical cohorts) on SZ case-control cohorts showed that cases had significantly lower cognitive polygenic scores compared to controls [40]. This finding was replicated later [37]. Using population-based samples, studies with different and robust methods have demonstrated a genetic negative correlation between SZ and cognition (g approach or cognitive subtests) [26,30,31,44,47,49,55,60,61,62,63,64,65,66]. Finally, in a large meta-analysis of 170 published twin and family heritability studies of more than 800.000 non-psychiatric and participants with SZ, substantial genetic overlap between cognitive phenotypes and schizophrenia liability was found, supporting partially shared genetic etiology [17].

Other studies have also shown a strong positive correlation between SZ genetic risk and EA, as previously mentioned [27,47,54,60,62,67,68,69,70] albeit few studies found non-significant or discrepant results [53,55]. Using the cFDR statistical approach, 10 gene loci were associated with SZ and EA/high EA, with effects in opposite directions [69]. A recent study using a PRS for EA was negatively associated to the risk of SZ [71].

#### 3.1.3. Causation Link 

Studies of genetic associations between schizophrenia and cognition provide evidence for shared genetic influence but causation is more difficult to prove, albeit recent studies address this caveat [28,65]. In particular, Toulopoulou et al. [65] explore the extent to which impairments in cognition mediate the influence of the PRS on schizophrenia liability, by using SZ PRS in causation models. They confirmed that the PRS alone can explain around 8% of inter-individual variation in schizophrenia risk, as previously demonstrated [7]. More than a third (2.7%) of this PRS influence would be mediated through cognition-related pathways. Moreover, almost 27% of the genetic vulnerability to schizophrenia is associated with cognition-related pathways not captured by the PRS. Finally, they estimate from their model that around a third of overall genetic risk of developing SZ is mediated through influences on cognition. 

#### 3.1.4. Findings from Cases and Sibling Studies

Recently, authors failed to demonstrate an association between SZ PRS and cognitive measures in a psychotic SZ sample [59]. The premorbid measure of intelligence and current cognitive functioning did not associate with the genetic risk of SZ. However, there was an association with poor cognition for the healthy sample. Authors question their sample sizes (unable to show an effect) and suggest that specific domains of cognition may be more closely and etiologically linked to schizophrenia than other domains. Yet, this differential effect could imply that factors other than the genetic risk of developing the disease play a significant role in determining the pathological trajectory of cognitive functions in patients. 

A recent study with a replication sample failed to show intermediate neurocognitive phenotypes in control samples with high PRS [72]. Interestingly, an intermediate phenotype was observed in sibling with high PRS (subthreshold psychotic phenotype, jumping to conclusion bias), while no cognitive dysfunction was observed nor in the sibling nor in the control sample, showing that in the absence of a sibling with psychotic disorder, expression of polygenic risk may even be protective against expression of psychosis proneness, as also reported by others [61] or constitute an advantage (e.g., for creativity, as reported by [52]). They thus hypothesize that higher rates of expression of psychosis intermediate phenotypes in sibling is not because of higher levels of genetic risk, but because of genetic risk interacting with higher rates of exposure to environmental risks in this group (shared environmental exposure with their affected relatives, for example urbanicity, childhood trauma, cannabis). Concerning the lack of association between neurocognition and SZ PRS in controls and siblings, authors suggest that variation in cognition as observed in schizophrenia may represent a ‘prognostic’ rather than a ‘disease’ factor [72]. Schizophrenia would represent the poorer outcome of a much broader psychotic phenotype, associated with cognitive alterations comorbidity. In other words, psychosis spectrum disorder may have a poorer outcome when it occurs in people who are at the lower end of the distribution of cognitive ability in the general population. As a result, schizophrenia genetic risk will predict lower cognitive ability in the general population (to a similar degree in controls and siblings, which is not intuitive). Thus, traditional measures of neurocognition may reflect the population distribution of cognitive ability impacting the prognosis of psychotic disorder (whereas cognitive biases may better index genetic risk according to this recent study).

This interpretation would explain the weak or negative results of the few studies that focused on the link between cognition and SZ PRS in SZ patients [39,59,73,74,75]. Albeit a study reports consistent results in small SZ sample [45], in the largest SZ sample with genetic and cognitive data to date, PRS for IQ and EA, but not for SZ (or BD), were associated with cognition [74]. Yet a study on a large sample of SZ patients, siblings and control reported that SZ PRS was associated with poorer performance on the block design task (spatial visualization), explaining 0.2% (*p* = 0.009) of the variance. But it is difficult to conclude on the effect of SZ PRS in the SZ sample, as authors performed their analyses on the whole sample [76].

All together, these latter findings suggest that in patients with schizophrenia, cognition is more strongly associated with polygenic risk that indexes cognitive traits in the general population than polygenic risk from mental disorders. This goes against the hypothesis that variation in cognitive impairment in schizophrenia is essentially a consequence of liability to the disorder, with greater impairment indicating greater liability. 

### 3.2. Meta-Analysis

Major findings are presented in Table 1. 

**Figure 1:**
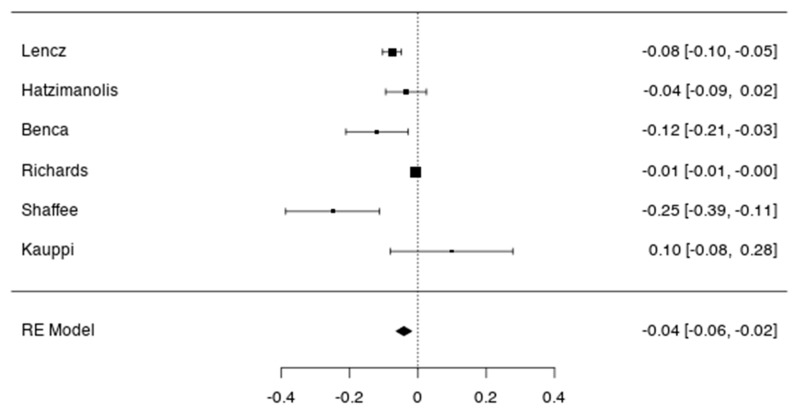
Forest plots of correlation between the genetic risk of schizophrenia and cognition in general and healthy participants. Error bars represent the 95% confidence intervals of the mean.

We found a significant and negative association between the genetic risk of schizophrenia and global cognition. The pooled effect size was −3.56 (*p* < 0.001, 95% CI: −0.064; −0.019) when the samples were relying on general and healthy participants (k = 6 studies, cf Figure 1). 

**Figure 2:**
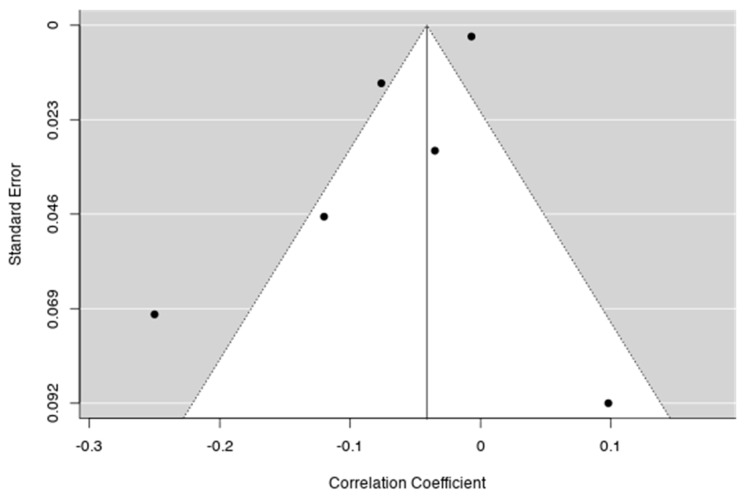
Funnel plot of the meta-analysis on general population and healthy samples. Each plotted point represents the standard error and the correlation coefficient between the SZ PRS and global cognitive functioning for a single study. The white triangle represents the region where 95% of the data points would lie in the absence of a publication bias. The vertical line represents the average correlation coefficient found in the meta-analysis.

When evaluating bias publication (Table 1), Fail-Safe N Analysis (File Drawer Analysis) provide *n* = 69,000, *p* < 0.001 (Fail-safe N Calculation Using the Rosenthal Approach), which is in favour of the robustness of the observed effect size (as 69,000 null studies would be needed to reduce the effect to non-significance). However, the classical measure of heterogeneity Cochran’s Q, as well as the I2 value for this subgroup suggests between-study variability (Table 1). With a small number of studies, we used the non-parametric correlation test for funnel plot asymmetry (Kendall’s Tau = −0.067, *p* = 1.000). The funnel plot is provided and shows the heterogeneity (Figure 2).

**Figure 3:**
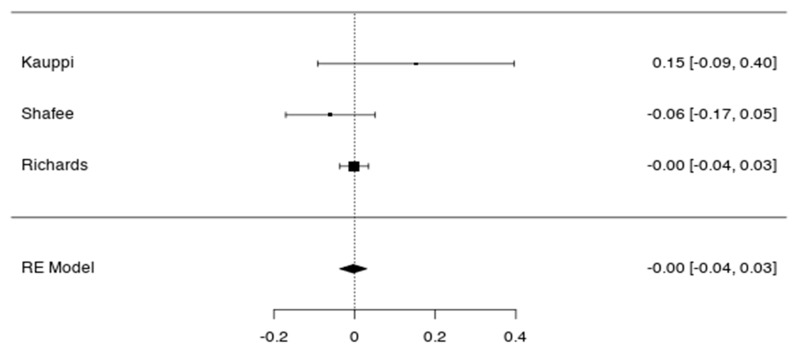
Forest plots of correlation between schizophrenia genetic risk and cognition in schizophrenia patients, Forest plot. Error bars represent the 95% confidence intervals of the mean.

**Figure 4:**
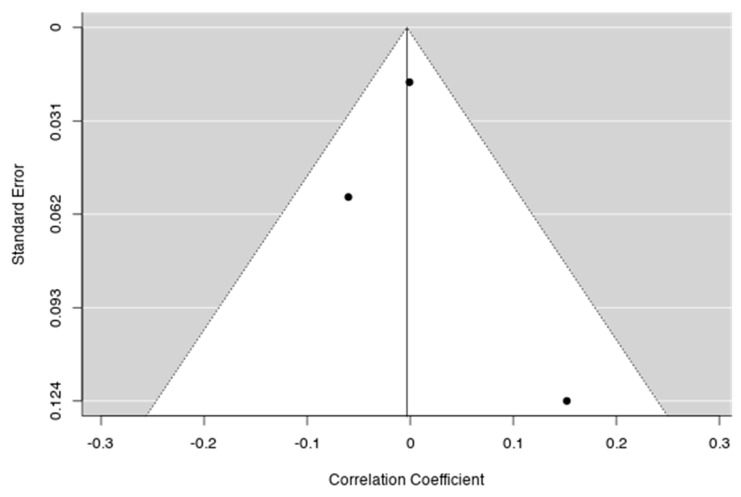
Funnel plot of the meta-analysis in schizophrenia patients. Each plotted point represents the standard error and the correlation coefficient between the SZ PRS and global cognitive functioning for a single study. The white triangle represents the region where 95% of the data points would lie in the absence of a publication bias. The vertical line represents the average correlation coefficient found in the meta-analysis.

On the other hand, no significant correlation was detected when the three studies devoted to schizophrenia patients were meta-analysed (*p* > 0.05) (Table 1, Figure 3). The funnel plots show that large statistic power tend to a null correlation coefficient (Figure 4). 

The publication bias has been evaluated with the rank correlation Test for Funnel Plot Asymmetry (Kendall’s Tau = 0.333, *p* = 1.000). The *I*^2^ statistic is more reliable than Q value in this case (unlike Q it does not inherently depend upon the number of studies considered), and shows no heterogeneity across studies.

## 4. Discussion

This article provides an overview of recent findings on genetic aspects underlying the link between cognitive functioning and schizophrenia and shows with a meta-analytic approach the differential effect of the genetic SZ risk on cognition in patients versus not. Analyses issued from different genetic tools converge toward the implication of the liability to schizophrenia in general cognition in population-based sample and in healthy controls. Cognitive deficit is reliably linked with inherited risk for schizophrenia. On the contrary, our review and meta-analysis could argue against universal pleiotropy for schizophrenia alleles and cognition, since cognition in SZ patients would be underpinned by the same genetic factors than in the general population, and substantially independent of common variant liability to the disorder. This up-to-date conceptualization of genetic aspects of neurocognitive functions in schizophrenia allows new therapeutic and research perspectives. Characterization of cognitive variations in population-based sample may indeed provide insights to inform the development of therapeutics for cognitive deficits in schizophrenia. 

However, there are several limits with the use of PRS to disentangle the links between SZ and cognition. Some are dependent of the caveats of the PRS technic (for review, see [77]). Some are more specific to this review. Although presence of family history is generally taken as an indicator of increased genetic risk, research suggests that molecular measures of Polygenic Risk Scores explain only around a fifth of the effect of family history, suggesting that a substantial part may be explained by epistasis and/or environmental effects and/or the interactions between genes and environment [78]. More specifically, the cumulative sum of risk associated alleles at common variants across the genome derived from the PGC genome-wide association study has been shown to account for around 7% of the variance in disease risk (Schizophrenia Working Group of the Psychiatric Genomics Consortium, 2014), which was corroborated later on [65]. Second, PRS are based on case–control comparisons, often with extreme cases, but is used in nonclinical sample (therefore not enriched for psychiatric symptoms). However, this reinforces the association between cognition and SZ PRS in our review. Third, in SZ patients PRS did not significantly predict any differences in symptoms or severity, or cognitive impairment up to now. This could be inherent to the clinical disease classification of SZ, which encompasses several different behavioural and cognitive traits that may not have identical genetic architectures. Besides, all current GWAS in schizophrenia rely on clinical manifestations, but not cognitive functions. GWAS analyses of specific SZ symptoms require very large sample sizes to be statistically well powered, and the currently available data sets on intensely phenotyped SZ patients are not yet large enough for this purpose. Fourth, as the PGC schizophrenia meta-analysis did not stratify on environmental risk (e.g., obstetrical complications, cannabis use), we cannot conclude which risk variant may be dependent on the environment, affecting individuals across their developmental stages. This could lead to the lack of association found in SZ patients. Finally, the meta-analyses provided in this article rely on a relatively small number of studies, yielding conclusions that could be easily altered with the addition of a few more studies. For the purpose of clarity, we provided every statistical tool to the reader, and analysed publication bias. However, this study is the first to provide a new and comprehensive insight on the differential impact of SZ PRS in participants with schizophrenia versus not. Finally, another important aspect is that schizophrenia is a heterogeneous disease, with possible distinct entities. Many studies point toward two groups with high/normal versus low cognitive functioning, possibly linked with different neurodevelopmental and environmental underpinnings [12]. The genetic study by [54] reveal that current SZ diagnoses aggregate over at least two disease subtypes: one part resembles high intelligence and bipolar disorder, while the other part is a cognitive disorder (independent of bipolar disorder). SZ PRS would then predict lower cognitive functioning in patients of the latter group (conflation between diagnosis and prognosis, as suggested by [72]).

### 4.1. The Cognitive Endophenotypes

Cognition remains an important endophenotype because delineating its underlying genetic mechanisms may identify promising targets for improving cognitive functioning in patients. Despite a lot of studies on the subject, it is difficult to draw a clear picture of which cognitive tests could be a good endophenotype for SZ. Using genetics and cognitive measures, few studies are replicated. In a SZ sample and in a population based sample, a genetic bilateral relationship was found between schizophrenia and performance IQ but not verbal IQ or other cognitive variables [37], which is concerning. Still, some authors found positive results for working memory [39,61,64] or for spatial visualization [76], verbal-numerical reasoning, reaction time and memory [44,47]. Executive functions (as latent variables) did not significantly relate to the SZ PRS [49]. It is indeed one of the less heritable cognitive domains [17]. It could be explained by the low test–retest reliability, secondary to their sensitivity to practice effect. In a recent study, endophenotype ranking score was used to rank candidate endophenotypes based on their heritability and genetic correlation with SZ [79]. Authors identified specifically two cognitive endophenotypes: CPT-IP and NART [80,81]. These cognitive measures were also genetically correlated with schizophrenia. CPT-IP is a measure of sustained attention and NART a measure of general cognitive ability, and have been consistently shown to share a genetic basis with schizophrenia [18], but rarely used in recent studies. This raises further interrogations. A better characterization of cognitive endophenotypes requires a homogenization of which cognitive subtests to use in SZ research studies, as suggested by recent groups [82]. Many molecular studies use traditional clinical and convenient neuropsychological test measures, which are often insensitive, non-specific, and ill-defined. Social cognition may also be useful, to better define subgroups of patients [46].

### 4.2. Perspectives

Our review and meta-analyses highlight the difficulties of determining how and to what extent the genetic of schizophrenia is specifically involved in cognitive functioning in SZ. When examining cognition at the gene levels, multiple genetic variants have been examined in different cognitive domains in schizophrenia but there have been few replication studies to date (for review, see [83]). The most examined candidate genes include *COMT, DISC1, HTR2A,* and *BDNF*, which all provided inconsistent findings and often associated with different aspects of cognitive dysfunction SZ. These variants were not all replicated in GWAS assessing the vulnerability to SZ, but the genetics implicated in cognition does not necessarily overlap with SZ vulnerability. If the same genes influence cognition in schizophrenia patients and the general population, which seems likely, the neural mechanisms regulated by these genes may nonetheless operate differently in patients. For example, these variants might interact with pharmacological treatments in SZ (as reported in [28] for *Dysbindin-1*). Indeed, all patients in above-mentioned studies are treated and there is no genetic study of drug naïve patients. Recent pharmacokinetic studies on SZ shed light on the individual variability in cognitive response to antipsychotic drugs, with a translational approach to transgenic mice models [28,29]. The authors underline that “the genetic variations predicting therapeutic response are not always linked to the causes of the pathology”, as in oncology [28].

Similar genetic basis in schizophrenia and non-psychiatric populations would not invalidate cognitive markers as endophenotypes of schizophrenia, since cognitive deficits in patients meet the criteria for endophenotypes [17]. It will be important to conduct comparative studies in large samples to determine whether the same or different genes and genetic variants influence cognition in schizophrenia patients and the general population. 

Using polygenic scores based on selected genetic risk variants clustering on specific functional pathways, rather than a broad selection of SNPs from the PGC, will be beneficial in the investigation of the specific effects that genetic risk factors for SZ have on brain function/structure and cognition. Future research should investigate whether a subset of common genetic variants (on a specific functional pathway) are more strongly involved. Recent studies using imagery have shed light on the possible structural pathways between genes and cognition [64,84], with one using a SZ PRS restricted to glutamate pathways [85]. The authors demonstrate the specific effects of a Glutamate-PRS on attention and brain activity, confirmed in a replication sample, suggesting a pathway specificity in the relationship between genetic risk for schizophrenia, the associated cognitive dysfunction and related brain processing. Another study allowed to use genetic variants “at risk” toward a functional and structural pathway, providing evidence to suggest that the CACNA1C risk variant rs2007044 (coding for a sub-unit of the calcium channel) is associated with poorer memory function that may result from cerebral disconnectivity among carriers [86]. These results are in line with the identified shared loci between cognitive functions and SZ [44], among other loci (with one located on chromosome 22q13.2, at a locus containing many genes including *TCF20, CYP2D6* and *NAGA*). The same team derivated a polygenic risk score from the PGC and the miR-137 downstream pathway (based on the set of 1016 genes whose expression was identified as being altered by miR-137 manipulation) [7,87] and demonstrated an impact on cognitive functioning in patients and controls. Others failed to show a strong involvement of hypothesis-driven PRSs [88], probably due to a lack of power. Finally, the role of inflammation and oxidative stress towards cognition in SZ patients was suggested, using a model of molecular pathway analysis on a large sample of patients with cognitive and genetic data [89].

In further studies, investigating interactions between other endophenotypes of schizophrenia and biological pathways that may be related to cognitive functions will be crucial.

## 5. Conclusions

In conclusion, current strategies hardly improve cognitive functioning in schizophrenia. A better understanding of genetic aspects of cognition in population and in patient samples is required. Largest samples with definition of homogenous subgroups and collaboration between task-forces will be needed to better understand how an endophenotype such as cognition will allow to tackle the disease pathway, from research and treatment perspectives. Genetic biomarkers such as PRS may help in the identification of these subgroups, which may reciprocally translate into clinical practice via precision medicine.

## Figures and Tables

**Table 1:** Meta-analyses of the association between the genetic risk of schizophrenia and global cognition in general and healthy participants (H) versus participants with schizophrenia (SZ).

**Meta-Analysis, Population**	**Random Effects Model (Hunter–Schmidt)**							
	**Estimate**	**se**		**Z**	**p**	**CI Lower Bound**		**CI Upper Bound**
H: k = 6, Intercept	**0.0413**	0.0116		−3.56	<0.001	−0.064		−0.019
SZ: k = 3, Intercept	−0.00327	0.0171		−0.191	0.848	−0.037		0.030
**Heterogeneity Statistics**								
	**Tau**	**Tau^2^**	***I*** **^2^**	***H*** **^2^**	***R*** **^2^**	**df**	**Q**	***p***
H	0.016	3 × 10^−4^ (SE = 0)	41.05%	1.696	.	5.000	42.697	<0.001
SZ	0.000	0 (SE = 6 × 10^−4^)	0%	1.000	.	2.000	2.601	0.272

H: Healthy sample; SZ: schizophrenia/psychotic sample; CI: 95% confidence interval.

## Supplementary Materials

The following are available online at https://www.mdpi.com/2077-0383/9/2/341/s1, Table S1: Molecular genetic approaches to test the overlap between cognition or educational attainment and schizophrenia: a review of the principal studies published the last 5 years.
