# Supplementary material for: Polygenic Risk Scores Shed Light on the Relationship between Schizophrenia and Cognitive Functioning: Review and Meta-Analysis
Source: J Clin Med. 2020 Jan 25;9(2):341. doi: 10.3390/jcm9020341 (PMC7074036; doi:10.3390/jcm9020341)
Supplement: Supplementary file 1 [file jcm-09-00341-s001.pdf]

Table S1: Molecular genetic approaches to test the overlap between cognition or educational attainment and schizophrenia : a review of the principal studies published the last 5years.				
**AUTHORS**	**DESIGN**	**Authors' principal results and conclusions**		**MAIN OUTCOME**
		**Genetic cognition factors on SZ**	**Genetic SZ factors on cognition**	
**GENERAL POPULATION SAMPLES**				
Lencz, 2014	PRS from a cognitive GWAS meta-analysis (N=4896, nine nonclinical cohorts comprising the COGENT) to four SZ case-control cohorts.	Cases had significantly lower cognitive polygenic scores compared to controls.	PRS for SZ (Pt<0.5) were associated with lower general cognitive ability (global R2 range from 0 to 2%, p=1.4*10-4)	General cognitive ability (G factor)
Hatzimanolis, 2015	- general population sample, male conscripts from 18-24yo(n = 1079, ASPIS)<br>-Follow-up genotyping in an internal sample (n = 738) + independent cohort (n = 825)<br>- Correlations with general cognitive ability and SZ PRS using GWAS results by the COGENT&PGC-SZ consortia	Sustained attention/vigilance and working memory were suggestively correlated with both COGENT and PGC-SZ derived polygenic scores (SZ PRS at Pt<.05 was correlated with attention/vigilance, r= 0.27, p=0.045 and spatial working memory, r=0.27, p=0.044, but not with other subtests or IQ (r =0.12)<br><br>"Common genetic variation explains some of the variability in neurocognitive functioning among young adults, particularly working memory, and provide supportive evidence that increased SZ genetic risk predicts neurocognitive fluctuations in the general population."		Cognitive subtests, general cognitive ability (non verbal IQ, Raven's matrice)
Bulik-Sullivan, 2015	- data from 24 GWAS<br>- cross-trait LD Score regression estimator of genetic correlations, to estimate 276 genetic correlations among 24 traits	Small Genetic correlation between SZ and Education attainment (R around 0.2, p<0.05)		EA
Davies, 2016	-UK Biobank, 500 000 individuals<br>- GWAs analyses for verbal-numerical reasoning, memory,		"The genomic regions (predicting cognitive scores and EA) include several loci, some of which have been associated with intracranial volume, neurodegeneration, Alzheimer's disease and schizophrenia."	Cognitive subtests<br><br>EA

	reaction time, EA			
Whitton, 2016	- dataset of Irish psychosis cases and controls (n = 1235)<br>-SZ risk SNPs in 8 candidate genes for cognitive deficiency		- "Strongest associations were for rs6984242 with both measures of IQ (p = 0.001) and episodic memory (p = 0.007). We link rs6984242 to CHD7 via a long range eQTL"<br>- These associations were not replicated in independent samples	IQ, working memory, episodic memory, attention
Liebers 2016	- 8616 older adults from the HSR Study, followed for an average of 10 years		SZ PRS (Pt 0.05) associated with decreased cognitive function (z = -3.00, p = .001 R2=0.04)(largely driven by an association with impaired attention and orientation(z = -3.33, P = 4.3×10^{-4}), but not with cognitive decline (contrary to Alzheimer PRS)- no association with MDD- or BD-PRS	Cognitive function (with the TICS), Cognitive subtests
Okbay, 2016	- GWAS for EA, discovery sample (n=101,069 to 293,723)+replication (n=111,349, UK Biobank)<br>- GWAS results for *EA* and published GWAS results for 14 other phenotypes, LD Score regression	"significant, but very small, genetic correlation of EA PRS with risk of SZ (r around 0.08, $p=3.2\times10^{-4}$)"		EA
Hagenaars, 2016	- UK Biobank; 502 655 community-dwelling participants<br>- reasoning, processing speed, memory<br>- GWAS (n= 112 151)<br>- LD regression, PRS		- Positive genetic correlation with EA (r=0.13, p=$1,1*10^{-16}$, n=111114) and negative genetic correlation with verbal-numerical reasoning (−0.30, p=$3.5*10^{-11}$, n= 36035), reaction time(-0.24,p=$2.1*10^{-9}$, n=111484), memory (-0.34,p=$1.1*10^{-16}$, n=112067)<br>- "SZ PRS positively associated with EA (*β*= 0.03)+ negatively associated with verbal-numerical reasoning (*β*=−0.06)."	Subtests<br>EA
Hubbard, 2016	- from 5109 to 5556 individuals (around 8 yo), ALSPAC cohort, grece)<br>- 29 415 schizophrenia cases and 40 101 control (GPC SZ) + CLOZUK (5554cases, 6299controls)	PRS for performance IQ was associated with increased risk for schizophrenia (*p*= $3.56E^{-04}$) in CLOZUK	Schizophrenia PRS was associated with lower performance IQ (R2=0.188, *p*= .001) and lower full IQ (R2=0.112, *P* = .013) in the general (young) sample but not with verbal IQ (R2=0.0305, p=0.194) nor in subtests<br>-Shared common genetic factors between schizophrenia and childhood cognitive ability	IQ, subtests based on their correspondence to MATRICS battery
Le Hellard, 2016	- 82315 SZ individuals from 49 non-overlapping case-control samples<br>- cFDR method to GWAS of SZ (n = 82 315), college completion (n = 95 427) ; years of	"polygenic overlap between SCZ and educational attainment (college completion and years of education)"<br>"10 gene loci associated with SZ and College completion and Years of education, with effects in opposite directions"		EA

	education (n = 101 069)			
Germine, 2016	~4300 individuals ages 8–21 years collected through the PNC<br>-Replication sample of ~700 individuals tested as part of the MGH		- "Replicable relationship between SZ PRS and speed of emotion identification<br>-Significant association between SZ PRS and speed of verbal reasoning<br>- These associations emerge relatively early in development<br>- No evidence of interaction between SZ PRS and age on neurocognitive performance"	Cognitive subtests
Krapohl, 2016	- 3152 unrelated adolescents from the UK-representative Twins Early Development Study, PRS		"SZ PRS (Pt 0.3)correlated positively with General Certificate of Secondary Examination English (R around 0.15 ($p<0.05$))<br>SZ PRS correlated negatively with Autism Quotient"	EA
Hill, 2016	- n= 53,949, 7 psychiatric disorders<br>- 2 GWAS cognition data in childhood+ older age, 1 GWAS on EA<br>- cross-trait LD score regression (300 genetic correlations, 25 traits)	- "negative genetic correlation between older age cognitive function (r= -.231, p = $3.81e^{-12}$) but not in childhood (r=-0.44, p=0.443)<br>- the correlation with EA, while not significant, was positive (r=0.06, p=0.093)<br>- the unique genetic variants responsible for cognitive variation in older age overlap with the polygenic component of schizophrenia, and in the case of childhood cognitive function, autism spectrum disorder"		General cognition,<br>EA
Nieuwboer, 2016	- GWIS method<br>- summary of differents GWAS	"analyses suggesting « that the previously reported genetic correlation between SZ and EA is probably induced by the observed genetic correlation between SZ and BD and the previously reported genetic correlation between BD and EA"		EA
Benca, 2016	- Sample of 452 individual twins (386 with all data) "Colorado LTS and CTS"<br>- PRS for 4 psychiatric disorders<br>- Three components of executive functions	- "IQ was negatively correlated with the SCZ PRS (Pt < $5*10^{-5}$)(r around -0.12), but this result did not survive correction for multiple testing"<br>- Latent variable of the executive functions not significantly relate to the SZ PRS (r around -0.1, ns)		Executive functions, IQ (from WAIS III and WASI)
Sniekers 2017	- combined GWAS data for intelligence in 78,308 unrelated individuals from 13 cohorts<br>Cross traits score	Of all 52 genes, seven (*CYP2D6, NAGA, NDUFA6, TCF20* and *SEPT3, FAM109B* and *MEF2C*) were implicated with schizophrenia		G factor
Trampush, 2017	- COGENT (data from 24 studies enrolling 35 298 individuals, mean age of 45.6 (s.d.±8.6) years)<br>- LD score regression	Negative correlation between general cognition factor g and SZ (r=-0.17, p= $4.09E^{10-4}$)		G factor
Blokland, 2017	- meta-analysis of 170 published twin and	- "Heritability estimates were comparable in nonpsychiatric and SZ samples"<br>- "genetic overlap between cognitive phenotypes and SZ liability overall relatively high (average r =−0.58;		Cognitive subtests

	family heritability studies of >800 000 nonpsychiatric and SZ subjects to accurately estimate heritability across many neuropsychological tests	SD = 0.22)"	
Lam, 2017	- large (N=107,207) GWAS of general cognitive ability (*g*) from COGENT, EA GWAS (Okbay et al 2016)<br>- LD-score regression	Negative genetic correlation (r=0.19, p<10-3) between SZ PRS and cognitive G factor	G factor
Riglin, 2017	-prospective population-based cohort study of 14701 children (ALSPAC) . PRS from PGC	"At age 7-9 years, SZ PRS showed associations with lower performance IQ (β -0·056, OR 1·13 [95% CI 1·04-1·23]), poorer social understanding (β -0·032, OR 1·08 [1·00-1·17]), worse language intelligibility and fluency (β -0·032, OR 1·10 [1·02-1·20]), more irritability (β 0·032, OR 1·07 [1·01-1·14]), and more headstrong behaviour (β 0·031, OR 1·08 [1·02-1·15])"	IQ, developmental domains
Smeland, 2017	- PGC cohort (n = 79 757 [cases, 34 486; controls, 45 271]); subtests in the UK Biobank cohort; general cognition in CHARGE (n = 53 949)+COGENT (n = 27 888).<br>- Genetic loci identified by cFDR analysis. Brain mRNA expression+ brain expression quantitative trait locus functionality	"-21 loci jointly influencing SZ and cognitive traits<br>- 2 loci shared between SZ and verbal-numerical reasoning, 6 loci shared between SZ and reaction time, and 14 loci shared between SZ and general cognitive function. One locus was shared between SZ and 2 cognitive traits and represented the strongest shared signal detected (nearest gene TCF20; chromosome 22q13.2), and was shared between SZ ($z = 5.01$; $p = 5.53 \times 10^{-7}$), general cognitive function ($z=-4.43$; $P = 9.42 \times 10^{-6}$), and verbal-numerical reasoning ($z=-5.43$; $p= 5.64 \times 10^{-8}$)<br><br>For 18 loci, schizophrenia risk alleles were associated with poorer cognitive performance.	Subtests, general cognitive function
Davies, 2018	- data from the CHARGE&COGENT consortia &UK Biobank (*N* = 300486)<br>-LD Score regression	Negative genetic correlation ($r=-0.23$, $p<10^{-3}$)	G factor
Savage, 2018	- GWAS from 14 cohorts (from children to adults)<br>- PRS, mendelian randomization, cross traits LD scores	"bidirectional causation with pleiotropic effects for SZ (suggesting that intelligence has a strong protecting effect on the SZ risk)"	G factor
Bansal, 2018	- GWAS results on EA (*n* = 363,502) and SZ (34,409 cases; 45,670	"strong genetic dependence between EA and SZ ($r2= 0.0612$, $p=4.4*10^{-4}$)that cannot be explained by chance, linkage disequilibrium, or assortative mating. Instead, several genes seem to have pleiotropic effects on EA and SZ, but without a clear pattern of sign concordance"-	EA

	controls). For replication and follow-up analyses, GRAS data<br>- series of statistical genetic analyses		
Luo,<br>2018	-Sample (discovery): 450 SZs+455 HCs ; Replication : 79 SZs and 87HCs<br>- 4522 SZ susceptible SNPs (according PGC), cognition (working memory)	"The modelization revealed a potential genetic-brain-cognition mediation pathway, indicating that polygenic risk factors could exert impact on phenotypic measures from brain structure to function, thus could further affect cognition in schizophrenia."	Subtests (working memory)
Brainstorm consortium, 2018	-25 brain disorders from GWASs of 265,218 patients and 784,643 HC ; 17 phenotypes from 1,191,588 individuals<br>-LD score regression	"- SZ showed mixed results, with a significantly negative correlation to intelligence (r= -0,1967, se= 0,0294, p= $2.07*10^{-11}$ (significance treshold p < 2.30 x 10^{-4})<br>(correlation with childhood cognitive performance : r=-0.03, SE=0.0539, p=0.4942 ; positive correlation to years of education (r= 0,0948, se=0.0231, p=0,000041826))	EA<br>IQ
Cordova-Palomera, 2018	- sample : 9000 individuals aged 8–22 years (PNC), 4183 included in the main analyses (Run of homozygoty, PRS, and cognition (WRAT))	"A higher SZ PRS is associated with higher cognitive performance across age (β = 0.027, SE = 0.011, p = 0.015)"	IQ
SØrensen, 2018	- case-cohort study on a population-based sample, 1470 individuals with SZ ; 7318 non SZ<br>- PRS_{SCZ} and PRS_{EDU} were calculated using discovery ES estimates from a meta-analysis of 34,600 cases, 45,968 controls& 293,723 individuals<br>-LD score regressions	"Higher PRS_{SCZ} increased the risk (incidence rate ratio [IRR]: 1.28; 95% CI : 1.19-1.36), whereas higher PRS_{EDU} decreased the risk of SZ (IRR, 0.87; 95% CI : 0.82-0.92) per standard deviation"<br>-PRS_{SCZ} and PRS_{EDU} was significantly negatively correlated (r = -.31, p < .01).	EA
Lam,<br>2019	Association analysis based on subsets (ASSET) (pleiotropic meta-analytic technique)	ASSET analysis revealed 235 independent loci associated with cognitive ability, education, and/or SZ at p < $5,3*10^{-8}$	General cognition, EA
Richards,	-Biobank Sample (as a	« SZ PRS (Pt 0.05) significantly predicted fluid intelligence in non-psychotic individuals in the Biobank	IQ

2019	replication sample)<br>-n =133 437, IQ-and SZ-PRS	sample ($p< 2.2 \times 10^{-16}$, ES = –0.137), though again with a smaller effect size than when using IQ PRS »		
Frei,<br>2019	- statistical tool, MiXeR (polygenic overlap irrespective of genetic correlation,<br>using GWAS summary statistics (including in PGC)	- MiXeR uncovers polygenic overlap between SZ and educational attainment. Despite a genetic correlation close to zero, the phenotypes share 8.3 K causal variants, while 2.5 K additional variants influence only educational attainment		EA
**SAMPLES INCLUDING SUBJECTS SZ SPECTRUM AND SIBLINGS**				
Kauppi 2015	- 63 SZ patients and 118 healthy controls		-PGRS was associated with decreased activation difference during a working memory task in control and in SZ patients, but did not correlate with any of the cognitive variable (p>.05)"<br>- Nb : The corresponding author has been contacted and provided these correlations between WASI and SZ PRS : HC : r=0,098 ; p=0.289, patients : r= 0,152 ; p=0,237	Subtest (working memory) and general cognition (from WASI)
Alloza,<br>2017	- sample of 28 SZ individuals+36 controls<br>-MRI, IQ	Regression analysis between IQ and SZ PRS (Pt 0.5) showed a tendency towards significance (on the whole sample : r =-0.742, p=0.052)		IQ (from WAIS)
Wang sh,<br>2018	-Sample=<br>1130 schizophrenia trios, taiwan sample<br>- SZ sample= "S-TOGET" sample<br>-SZ PRS from GPC		"SZ PRS (Pt 0.1) explained about 0.5% of variability for cognitive performance in patients, and the estimated R-square is similar to that reported in general population- Increasing genetic risk of SZ was associated with poor neurocognitive performance". In the SZ sample, R2 =0.47% p=0.03	Subtests (WSCT, CPT), general latent variable (via SEM)
Shafee,<br>2018	- genome-wide SNP data from 314 psychotic (Psych) and 423 healthy research participants in the Bipolar-Schizophrenia Network for Intermediate Phenotypes (B-SNIP) Consortium<br>+<br>more than 4500 (young= around 13.8yo) healthy subjects from the PNC		- In healthy individuals from B-SNIP : SZ PRS associated with lower BACS scores ($r = -0.17$, $p = 6.6 \times 10^{-4}$ at $P_T = 1 \times 10^{-4}$, n=423), but not with WRAT or EY.<br>- In healthy individuals from the PNC cohort : no association<br><br>- In psychotic sample: SZ PRS did not associate with variations in any of these three phenotypes (r=-0.06, p=0.31 (n = 314))<br><br>- In the NPFAM group : (n=243 (siblings) : r =-0.16, p = 0.02) and in HC (n=180) : r=-0.25, p= 1.9x10-3)	measure of current cognitive abilities (the Brief Assessment of Cognition in Schizophrenia, BACS)<br><br>EA<br><br>Premorbid intelligence (WRAT)
Ranlund,<br>2018	- Sample n=4442 from Europe: 1,087 SZ patients, 822 unaffected first-degree relatives;		"SZ PRS associated with poorer performance on the block design task (n=3089 among 4442 participants)(spatial visualization)+ explained 0.2% (p = 0.009) of the variance. This measure is an endophenotype for SZ with shared genetic risk variants (data adjusted with clinical group as a	Subtests

	2,333 controls.<br>- Cognition (block design, digit span, Ray Auditory Verbal Learning Task) ; PRS from PGC		covariable)”	
Nakahara, 2018	- Sample(US) : 127 SZ individuals+136 healthy volunteers<br>- MATRICS cognitive assessment, PRS from PGC , whole genome analyse		-“SZ PRS showed significant negative correlations with each cognitive domain- Genome-wide association analyses identified loci associated with attention/vigilance, verbal memory and reasoning/problem solving”	Subtests, MATRICS
Touloupoulou, 2019	-CBDB Sample: 1313 members of 1078 families: 416 patients with SZ,290 unaffected siblings, and 607 controls.<br>- modeling (general cognition and SZ-PRS as latent traits)		- “SZ-PRS explained 8.07% [(CI) 5.45–10.74] of SZ risk in the sample. Of this, more than 1/3 (2.71% (CI 2.41–3.85) of the PRS influence was mediated through cognition paths, exceeding the direct influence of SZ-PRS on SZ risk (1.43%(CI 0.46–3.08)). The remainder of the PRS influence (3.93% (CI 2.37–4.48))) reflected reciprocal causation between SZ liability and cognition (e.g. mutual influences in a cyclical manner)”<br>- Analysis of genetic variance components of SZ liability indicated that 26.87% (CI 21.45–32.57) was associated with cognition-related pathways not captured by PRS.<br>- correlation between SZ-PRS and g factor : r = -0.297, p<.001<br>- In authors’model, 33.51% (CI 27.34–43.82%) of overall genetic risk is mediated through influences on cognition	General cognition (as a latent trait)
Van os, 2019	- two samples for discovery (“GROUP” : n = 336 controls; 649 siblings) + replication “EUGEI” (n = 1208 controls ; 1106 siblings)<br>- cognitive evaluation, including the « jumping to conclusion bias »		-“ Association between PRS (Pt 0.05) and Jumping to conclusion bias was only associated with PRS in the sibling group ($p<10^{-3}$ versus p=0.08).”<br><br>- “Cognitive ability was weakly negatively and non-significantly associated with PRS in both the sibling (p=0.36) and the control group (p=0,83) in the discovery sample, and weakly in the healthy group (replication sample, p=0.02)”	Global cognitive score Tscore derived from z-scores of 4 WAIS III subtests
Richards, 2019	- genetic and cognitive data in 3034 SZ cases from 11samples<br>- cognition and EA		“PRS for both population IQ (ES= 0.199, p= $4.39X10^{-28}$) and EA (ES=0.188, p= $1.27x10^{-26}$) were positively correlated with cognition in SZ.<br>In contrast, there was no association between cognition in SZ cases and SZ-PRS (p= .39), BD (p= .51), or MDD (p= .49). No individual variant approached genome-wide significance in the GWAS.”<br>(link between SZ-PRS and G factor : ES= -0.017, SE=0.019, p=0.386)	G factor, EA

PRS, Polygenic risk score ; SZ, schizophrenia ; GWAS, Genome Wide Association Study; Pt, p-value tresholds used for constructing polygenic scores; EA, Educational attainment ; IQ, Intellectual Quotient; CI, Confidence Interval; MATRICS, MATRICS Consensus Cognitive Battery; ES, Effect Size; SD, Standard Deviation ; BD, Bipolar Disorder; MDD, Major depressive disorder ; PGC, Psychiatric Genomics Consortium ; cFDR method, conditional False Discovery Rate method; LD, Linkage Desequilibrium
